# Development and Validation of Prognostic Nomogram for Primary Peritoneal Serous Carcinoma Compared With FIGO Staging System: A Population-Based Study

**DOI:** 10.3389/fonc.2021.651969

**Published:** 2021-08-19

**Authors:** Ming Chen, Zhenzhen Wen, Zhengwei Qi, Min Gao

**Affiliations:** Department of Gastroenterology, Sir Run Run Shaw Hospital, College of Medicine Zhejiang University, Hangzhou, China

**Keywords:** primary peritoneal serous carcinoma (PPSC), nomogram, FIGO staging system, overall survival, SEER

## Abstract

**Background:**

Primary peritoneal serous carcinoma (PPSC) is a rare tumor that lacks a prognostic prediction model. Our study aims to develop a nomogram to predict overall survival (OS) of PPSC patients.

**Methods:**

Patients confirmed to have PPSC between 2004 and 2012 were selected from the Surveillance, Epidemiology, and End Results (SEER) database. LASSO and multivariate Cox regression analyses were used to screen for meaningful independent prognostic factors to construct a nomogram model for 3-, 5-, and 10-year OS among patients with PPSC. The nomogram compared the discrimination, calibration, and net benefits with the International Federation of Gynecology and Obstetrics (FIGO) staging system of PPSC patients.

**Results:**

Eight variables were selected to establish the nomogram for PPSC. The established nomogram performed significantly better than the FIGO staging system (*p* < 0.05). The 3-, 5-, and 10-year OS of PPSC was 0.498, 0.306, and 0.152, respectively. Patients of old age, widowed marital status, grade high, FIGO IIIB, IIIC, or IV, lymph node metastasis, no lymphadenectomy, no surgery, and no chemotherapy got higher score which corresponds with higher risk and lower OS. In the multivariate Cox regression analysis, age, histological grade, FIGO staging, lymph node metastasis, and lymphadenectomy (four or more) were identified as independent prognostic factors for PPSC.

**Conclusions:**

PPSC patients have distinct characteristics with respect to their presentation and survival outcomes. A prognostic nomogram constructed by various clinical indicators can provide better and more accurate predictions for patients with PPSC.

## Introduction

Primary peritoneal cancer (PPC) is a kind of extraovarian malignant tumor that widely distributes in the peritoneal cavity, mainly on the surface of the omentum and peritoneum with intact ovaries or minimal ovarian involvement (1). PPC first described by Swerdlow in 1959 is seen predominantly in elderly and postmenopausal women (2, 3). The incidence rates for primary ovarian and peritoneal cancers are 5.08 and 0.65 per 100,000 respectively in the US population (4). Primary peritoneal serous carcinoma (PPSC) approximately accounting for 90% of PPC, is histologically, molecularly, and clinically similar to stage III/IV epithelioid ovarian serous carcinoma (5). Unfortunately, there is no standard treatment for PPSC while it is traditionally treated in a similar way to stage III/IV epithelioid ovarian serous carcinoma, including maximum surgical removal of peritoneal deposits followed by platinum/taxane chemotherapy regimens (6, 7). The survival rate of patients with PPSC is similar to or 2 to 6 months less than that of ovarian cancer patients (6, 8, 9).

The highest numbers in PPSC occurred in the 65–69 age group (4). It is estimated that the group of people 65 years and older will comprise 20.1% of the US population by 2030 (10). These statistics outline the significance of an increasingly older cancer population that will require oncologic management specific for their needs. While the number of elderly women living in the USA is expected to increase, there is very little data on the survival and prognosis of elderly women diagnosed with PPSC (11). Currently, most previous studies on PPSC have insufficient sample size, incomplete clinical data, short follow-up time, or single-center deviation, and no study has separately investigated the potential prognostic variables in patients of PPSC. The International Federation of Gynecology and Obstetrics (FIGO) staging system is used to evaluate the prognosis of PPSC (7). However, no study has compared and analyzed the effectiveness of FIGO staging system for patients of PPSC. Therefore, we conducted a large, population-based, long-term review, and analysis of clinicopathological and treatment data of PPSC from the Surveillance, Epidemiology, and End Results (SEER) database.

## Materials and Methods

### Patient Selection

Women diagnosed with PPSC between 2004 and 2012 were initially identified from the SEER database. The inclusion criteria were as follows (1): the primary site label was “C48.1-specified parts of peritoneum” or “C48.2-peritoneum, NOS” (2); the International Classification of Diseases (ICD) code O-3 morphology was 8441/3, 8460/3, or 8461/3 (3); PPC as the only or first primary tumor confirmed by histology; and (4) active follow-up to ensure reliable patient status. The exclusion criteria were as follows (1): missing information on race, marital status, histological grade, lymph node status, FIGO staging system based on the American Joint Committee on Cancer (AJCC) TNM staging system, surgery type, lymphadenectomy, or chemotherapy (2); patients died within 1 month or were followed up less than 1 month since initial diagnosis.

### Cohort Definition and Variable Recode

The patients diagnosed between 2004 and 2009 were randomly divided into the training and internal validation cohorts while the patients diagnosed between 2010 and 2012 were as an external validation cohort. The training cohort was used to screen variables and construct the prediction model. The internal and external validation cohorts were used to validate the results obtained by the training cohort.

Surgery types included (1) none (2), local tumor excision (3), simple/partial surgical removal of primary site (4), total surgical removal of primary site (5), surgery stated to be “debulking,” and (6) radical surgery (partial or total removal of the primary site with partial or total removal of other organs). The FIGO staging system was based on AJCC cancer staging manual (8th edition) (12) (1): Stage IIIA2: T3a-NX/NO/N1-MO (2), Stage IIIB: T3b-NX/NO/N1-MO (3), Stage IIIC: T3c-NX/NO/N1-MO, and (4) Stage IV: TX-NX-M1.

### Statistical Analysis

Continuous and categorical data are expressed as frequencies with percentages. A chi-square test was performed to explore the relationship between the clinical features of different groups. The optimal cutoff values of age were assessed by the X-tile software. Analysis items with *p* < 0.05 were considered statistically significant. LASSO and multivariate Cox regression analyses were performed for all variables, and variables with *p* < 0.05 in multivariate Cox regression were identified as independent risk factors. The chi-square test and Cox regression analysis with 95% confidence interval (CI) and hazard ratios were calculated.

A nomogram was formulated by the clinical and statistical significance of multivariate analysis using R version 4.0.3 (http://www.r-project.org/). Overall survival (OS) was the endpoint of interest in this study, calculated from diagnosis to death of all causes or to date of last follow-up in November 2017. Patients who were alive at the last follow-up were censored. The 3-/5-/10-year OS were estimated by the prediction model. The nomogram was validated both internally and externally. The area under the time-dependent receiver operating characteristic curve (AUC) calculated by bootstrapping was used to evaluate discriminative ability. Generally, an AUC value that is greater than 0.7 indicates a reasonable estimation. Calibration plots were used to evaluate calibrating ability. Compared with the FIGO staging system, the decision curve analysis (DCA), integrated discrimination improvement (IDI), net reclassification index (NRI), and likelihood ratio test (LR test) were used to evaluate the clinical benefits and practicality of the nomogram. DCA is a method to evaluate the clinical benefit of alternative models and is applied to nomograms by quantifying the net benefit under different threshold probabilities (13). The curves of all patient treatment plans (representing the highest clinical cost) and no treatment plans (representing no clinical benefit) are drawn as two references (14). IDI and NRI are two alternative methods of AUC, used to evaluate the improvement of risk prediction and measure the effectiveness of the new model (15, 16). In statistics, LR test is a statistical test used to compare the goodness of fit of two statistical models (null model and alternative model). The test is based on a likelihood ratio, which indicates how many times more likely the data are under one model than the other. Then, you can use the likelihood ratio or the equivalent logarithm to calculate the *p*-value, or compare it with a critical value, to decide whether to reject the null model (17). The Kaplan-Meier OS curves were used to test the discrimination of variables.

## Results

### Characteristics of Patients and Disease

A total of 691 patients diagnosed as PPSC between 2004 and 2009 were enrolled as a developmental cohort and randomly divided into a training cohort and an internal validation cohort by a ratio of 7:3. Meanwhile, 292 patients diagnosed as having PPSC between 2010 and 2012 were enrolled as an external validation cohort. The basic characteristics of patients are listed in **Table 1**. The training and internal validation cohorts had no significant difference (*p* > 0.05). Patients of age between 53 and 76 (74.0% *vs*. 70.5%), white ethnicity (91.2% *vs*. 87.0%), married women (59.8% *vs*. 62.7%), poorly differentiated carcinoma (50.8% *vs*. 45.6%), and FIGO Stage IV (57.9% *vs*. 49.2%) constituted the majority of both the developmental and external cohorts. However, the whole population had a relatively low rate of lymph nodes metastasis (27.2% *vs* 25.7%) in the developmental and external cohorts. Moreover, most of patients did not receive lymphadenectomy (55.0% *vs* 54.8%). Debulking surgery (53.7% *vs* 63.7%) was the main type of surgery underwent by PPSC patients and about 90% of patients received chemotherapy (86.7% *vs* 90.8%) in the developmental and external cohorts. The median follow-up time was 117 months (95% CI: 108–125) in the developmental cohort while 63 months (95% CI: 59–67) in the external validation cohort, respectively (*p* < 0.001). The median survival time was 38 months (95% CI: 35–41) in the developmental cohort and 36 months (95% CI: 32–39) in the external validation cohort, respectively (*p* = 0.812).

**Table 1:** Baseline demographic and clinical characteristics of patients with PPSC.

Characteristics	Training cohort	Internal validation cohort	*p*-Value	Total developmental cohort (2004–2009)	External validation cohort (2010–2012)	*p*-Value
***N***	487	204		691	292	
**Age**			0.404			0.317
21–52	55 (11.3%)	26 (12.7%)		81 (11.7%)	33 (11.3%)	
53–76	354 (72.7%)	157 (77.0%)		511 (74.0%)	206 (70.5%)	
77–91	78 (16.0%)	21 (10.3%)		99 (14.3%)	53 (18.2%)	
**Race**			0.722			0.076
White	44 6(91.6%)	184 (90.2%)		630 (91.2%)	254 (87.0%)	
Black	13 (2.7%)	5 (2.5%)		18 (2.6%)	15 (5.1%)	
Other	28 (5.7%)	15 (7.3%)		43 (6.2%)	23 (7.9%)	
**Marital status**			0.830			0.627
Single	50 (10.3%)	22 (10.8%)		72 (10.4%)	32 (11.0%)	
Married	287 (58.9%)	126 (61.8%)		413 (59.8%)	183 (62.7%)	
Divorced/separated	63 (12.9%)	25 (12.2%)		88 (12.7%)	29 (9.9%)	
Widowed	87 (17.9%)	31 (15.2%)		118 (17.1%)	48 (16.4%)	
**Histological grade**			0.977			0.195
Low	26 (5.3%)	11 (5.4%)		37 (5.4%)	10 (3.4%)	
High	461 (94.7%)	193 (94.6%)		654 (94.6%)	282 (96.6%)	
**FIGO staging**			0.660			<0.001
IIIA2	17 (3.5%)	11 (5.4%)		28 (4.0%)	16 (4.5%)	
IIIB	30 (6.2%)	14 (6.8%)		44 (6.4%)	21 (6.6%)	
IIIC	154 (31.6%)	65 (31.9%)		219 (31.7%)	171 (39.7%)	
IV	286 (58.7%)	114 (55.9%)		400 (57.9%)	84 (49.2%)	
**Lymph nodes**			0.223			0.622
N	348 (71.5%)	155 (76.0%)		503 (72.8%)	217 (74.3%)	
Y	139 (28.5%)	47 (24.0%)		188 (27.2%)	75 (25.7%)	
**Lymphadenectomy**			0.574			0.421
N	263 (54.0%)	117 (57.4%)		380 (55.0%)	160 (54.8%)	
1–3	60 (12.3%)	20 (9.8%)		80 (11.6%)	42 (14.4%)	
4 or more	164 (33.7%)	67 (32.8%)		231 (33.4%)	90 (30.8%)	
**Surgery**			0.904			<0.001
N	30 (6.2%)	8 (3.9%)		38 (5.5%)	5 (1.7%)	
Local	8 (1.6%)	3 (1.5%)		11 (1.6%)	0 (0.0%)	
Partial	25 (5.1%)	12 (5.9%)		37 (5.4%)	11 (3.8%)	
Total	13 (2.7%)	5 (2.4%)		18 (2.6%)	14 (4.8%)	
Debulking	260 (53.4)	111 (54.4%)		371 (53.7%)	186 (63.7%)	
Radical	151 (31.0%)	65 (31.9%)		216 (31.2%)	76 (26.0%)	
**Chemotherapy**			0.596			0.074
N	67 (13.8%)	25 (12.3%)		92 (13. 3%)	27 (9.2%)	
Y	420 (86.2%)	179 (87.7%)		599 (86.7%)	265 (90.8%)	
**Survival outcome**			0.763			<0.001
Dead	408 (83.8)	169 (82.8)		577 (83.5)	213 (72.9)	
Censor	79 (16.2)	35 (17.2)		114 (16.5)	79 (27.1)	
**Median follow-up time**	114 (104–124)	120 (109–131)	0.679	117 (108–125)	63 (59–67)	<0.001
**Median survival time**	34 (30–38)	42 (34–49)	0.301	38 (35–41)	36 (32–39)	0.812

FIGO, the International Federation of Gynecology and Obstetrics.

### Nomogram Variable Screening

**Figure 1:**
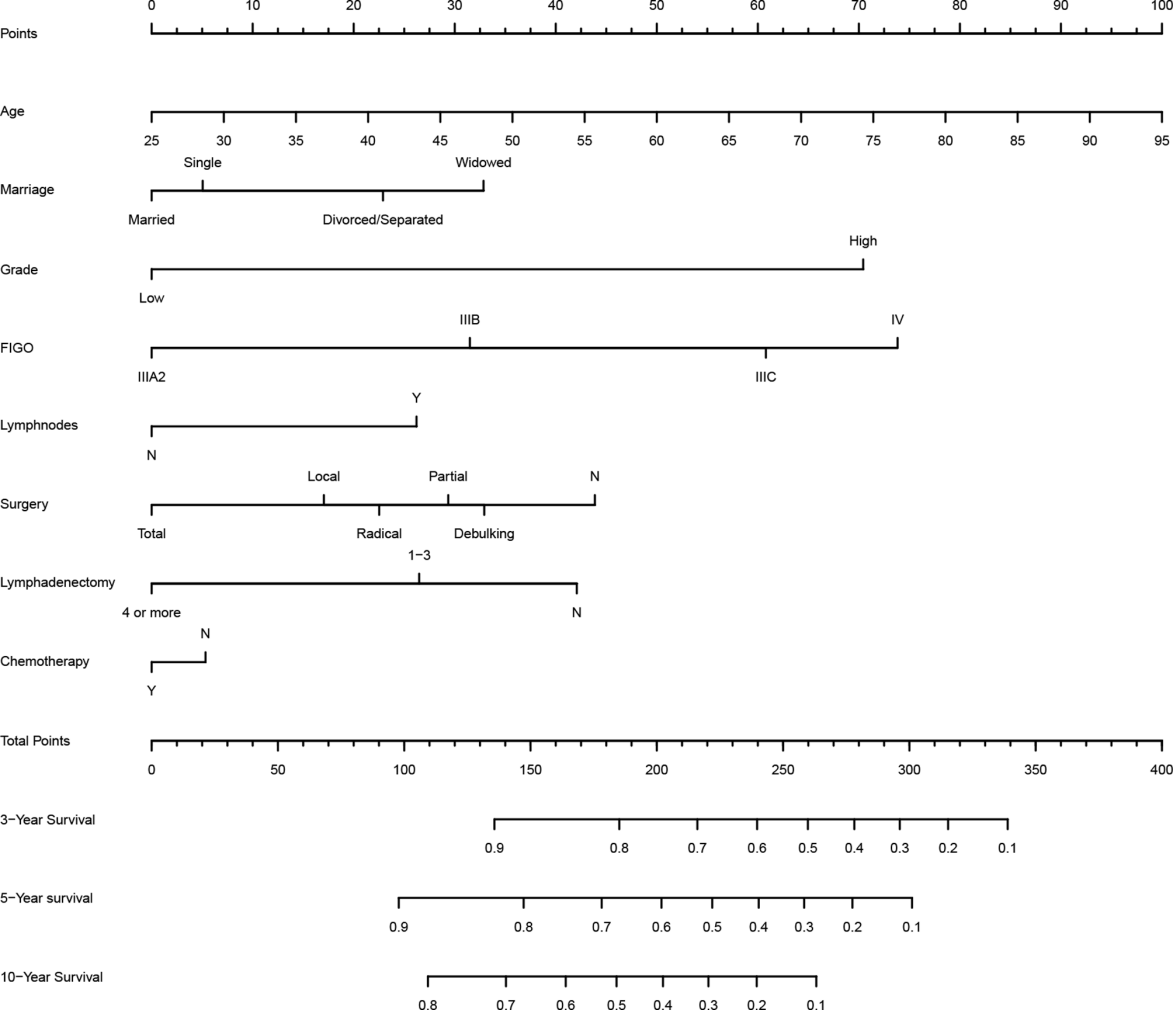
Survival nomogram to predict the 3-, 5-, and 10-year OS of PPSC patients.

According to LASSO and stepwise regression results, the model containing age, marital status, histological grade, FIGO staging, lymph nodes metastasis and lymphadenectomy had minimal AIC value in the training cohort. Nevertheless, it is important to consider both clinical and statistical significance when selecting variables for inclusion (18). Therefore, we added surgery type and chemotherapy into the prediction model (**Figure 1**), because they are the main treatment correlated with prognosis in clinical practice for PPSC patients. In the multivariate Cox regression analysis, age, histological grade, FIGO staging, lymph node metastasis and lymphadenectomy (four or more) were identified as independent prognostic factors for PPSC (**Table 2**). As the nomogram showed (**Figure 1**), every variable had a corresponding nomogram score listed in the **Table 2**.

**Table 2:** Multivariate Cox regression analysis and nomogram score of variables in the nomogram.

Variable	Multivariate analysis			Nomogram score
	HR	95% CI	*p*-Value	
**Age**	1.022	1.011–1.033	<0.001	1.4* (Age–25)
**Marital status**				
Single	1			5
Married	0.926	0.662–1.297	0.655	0
Divorced/separated	1.311	0.864–1.989	0.203	23
Widowed	1.525	1.023–2.273	0.038	33
**Histological grade**				
Low	1			0
High	2.928	1.598–5.367	<0.001	70
**FIGO staging**				
IIIA2	1			0
IIIB	1.568	0.729–3.376	0.219	31
IIIC	2.481	1.258–4.893	0.008	61
IV	3.019	1.546–5.896	<0.001	74
**Lymph node metastasis**				
N	1			0
Y	1.489	1.141–1.942	0.003	26
**Lymphadenectomy**				
N	1			42
1–3	0.789	0.573–1.087	0.147	26
4 or more	0.528	0.4004–0.696	<0.001	0
**Surgery**				
N	1			44
Local	0.665	0.249–1.774	0.415	17
Partial	0.802	0.434–1.482	0.482	29
Total	0.514	0.237–1.115	0.092	0
Debulking	0.847	0.552–1.299	0.446	33
Radical	0.723	0.458–1.143	0.165	23
**Chemotherapy**				
N	1			5
Y	0.919	0.681–1.240	0.596	0

FIGO, the International Federation of Gynecology and Obstetrics. *means multiply by.

### Nomogram Validation

**Figure 2:**
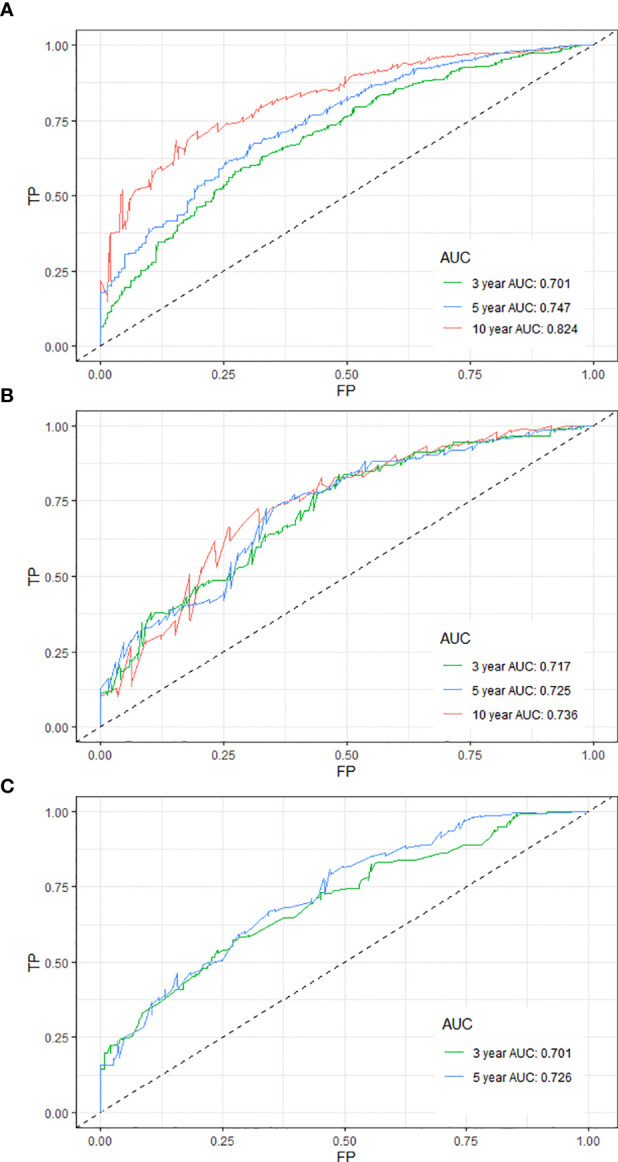
Time-dependent receiver operating characteristic curves for predicting the 3-, 5-, and 10-year OS of PPSC patients in the training **(A)**, internal validation **(B)**, and external validation **(C)** cohorts.

The time-dependent receiver operating characteristic curves were generated to further evaluate the predictive performance for 3-, 5-, and 10-year OS (AUC = 0.701, 0.747, and 0.824 in the training cohort and AUC = 0.717, 0.725, and 0.736 in the internal validation cohort, respectively) (**Figures 2A, B** **)**. Due to shorter follow-up time in the external validation cohort, we only calculated the predictive performance for 3- and 5-year OS (AUC = 0.701 and 0.726, respectively) (**Figure 2C**). All the AUC values are greater than 0.7 for the prediction of OS in both the training and validation cohorts, indicating favorable discrimination by the nomogram. Additionally, the calibration curves for the probability of 3-, 5-, and 10-year survival exhibited an optimal agreement between the predicted and observed OS (**Supplementary Figure 1**). As above, the survival nomogram for prediction of PPSC patients had considerable discriminative and calibrating abilities.

### Clinical Value of the Nomogram Compared With the FIGO Staging System

Additionally, DCA curves showed that the nomogram added more net benefits than the FIGO staging system (**Supplementary Figure 2**). The changes in IDI and NRI indicated that the nomogram had greater accuracy for predicting prognosis of PPSC patients than the FIGO staging system (**Supplementary Table 1**). LR test was also used for comparing the goodness of fit of the survival nomogram and the FIGO staging system, which further proved the effectiveness of the nomogram (**Supplementary Table 1**).

### Clinical Example

**Supplementary Figure 3** shows screenshots of the updated web calculator with a clinical example which can be found at https://peritoneal-carcinoma.shinyapps.io/peritoneal-carcinoma/. Clinicians can log in to the website and enter patient characteristics to automatically predict prognosis of PPSC patients.

## Discussion

We structured a survival nomogram predicting 3-, 5-, or 10-year survival more accurately than the FIGO staging system maybe a useful tool to better predict prognosis and optimize therapeutic regimes for patients with PPSC. The median follow-up time was 117 months (95% CI: 108–125) and 83.5% (577/691) of patients were observed ending event during follow-up. Therefore, these data could well predict the OS of PPSC patients within 10 years. The 3-, 5-, and 10-year OS of PPSC patients was 0.498 (standard error (SE): 0.019), 0.306 (SE: 0.018), and 0.152 (SE: 0.015), respectively. The median survival time was 38 months (95% CI: 35–41) longer than most of the previous studies (2, 3, 6, 19–23), which may be due to the longer follow-up time in this study.

In published nomograms, the range of variables considered is usually based on data availability and clinical evidence rather than on statistical significance (18). Surgery and chemotherapy are the main therapies for PPSC patients. Given their clinical significance, they should be added into the prediction model. The effectiveness of the nomogram had been verified by the internal and external validation cohorts. From the nomogram, we found that patients of old age, widowed marital status, grade high, FIGO IIIB, IIIC, or IV, lymph node metastasis, no lymphadenectomy, no surgery, and no chemotherapy got higher score which corresponds with higher risk and lower OS. Measured by standard deviation along nomogram scales, age, histological grade, and FIGO staging system were the top 3 prognostic factors, followed by lymphadenectomy and surgery type (**Figure 1**). To use the nomogram, each variable of a patient has a specific point based on specific value. The sum of the points of all variables is the total points of the patient, and finally the corresponding 3-, 5-, and 10-year OS is found (**Figure 1**). In order to make it more convenient for clinicians to use, you can log in to the website (https://peritoneal-carcinoma.shinyapps.io/peritoneal-carcinoma/) and enter patient characteristics to automatically predict OS of PPSC patients (**Supplementary Figure 3**).

**Figure 3:**
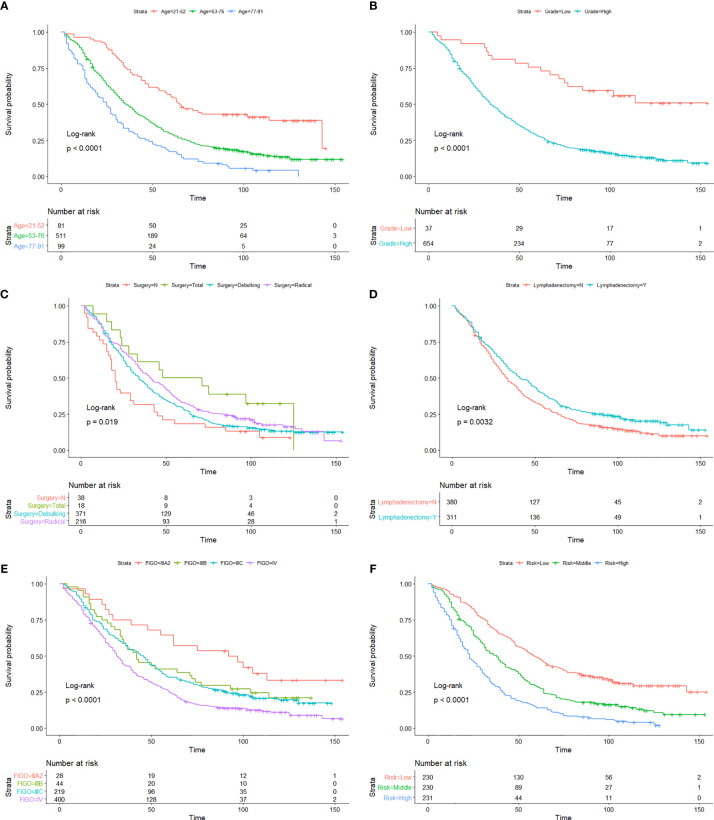
Kaplan-Meier OS curves of age **(A)**, histological grade **(B)**, surgery type **(C)**, lymphadenectomy **(D)**, FIGO staging system **(E)**, and risk stratification **(F)** in the developmental cohort.

As we can see in the nomogram, OS decreases with age for PPSC patients. Multivariate analysis revealed that age was an independent prognostic factor of PPC patients. The X-tile software used the ages of 52 and 76 as the cutoff points, which could better distinguish the OS (**Figure 3A**). The median age was 65 years old and about 54.4% patients were over 65 years old in this study similar with Bloss’s and Eltabbakh’s studies (3, 23). The 3-, 5-, and 10-year OS of PPSC patients under 65 years old was 0.577 (SE: 0.028), 0.306 (SE: 0.027), and 0.152 (SE: 0.025), respectively. However, the 3- and 5-year OS of PPSC patients over 65 years old was only 0.433 (SE: 0.026) and 0.104 (SE: 0.016), respectively, which was significantly decreased. Unfortunately, the 5-year OS of PPSC patients over 65 years old was only one-third of the 5-year OS of PPSC patients under 65 years old and even lower than the 10-year OS of PPSC patients under 65 years old. The poor prognosis of elder PPSC patients may be mainly due to the high incidence of medical comorbidities, poor basic conditions, inability to tolerate surgery and chemotherapy, and lack of active treatment options in an older and more common terminal disease population (11, 24–27). In consequence, with increasingly aging of population, more researches are urgently needed to improve the treatment of elderly patients of PPSC.

There is increasing evidence that histology plays an important role in overall patient’s prognosis (28–31). Currently, the histological grade of PPSC refers to the two-level grading system for ovarian serous carcinomas proposed by Malpica et al. of M.D. Anderson Cancer Center (MDACC), consisting of low grade and high grade (7, 32, 33). The binary grading system for serous carcinoma is based primarily on the assessment of nuclear atypia with the mitotic count used as a secondary criterion. Traditionally, serous carcinomas have been graded as well, moderately, or poorly differentiated. Low-grade serous carcinomas in this system have a high correlation with grade 1 tumors, and high-grade serous carcinomas have a high correlation with grades 2 and 3 tumors in the Shimizu–Silverberg and the FIGO grading systems. There is very little data on the effect of histological grade on the prognosis of PPSC. In this study, the average ages of the low- and high-grade patients are 52.9 and 65.4 years old, and the median survival times of the low- and high-grade patients are 114 and 34 months (**Figure 3B**), respectively. The major clinical features of low-grade PPSC were similar to the previous research published by Gershenson et al. (34, 35), including relative young age at diagnosis, prolonged OS, and relative chemoresistance. Unfortunately, about 94.6% of PPSC patients were diagnosed of high grade with older age and poor prognosis in this study.

Many authors believed that optimal cytoreduction could significantly improve prognosis of PPC patients (23, 36, 37). However, PPC is generally of multifocal and multiclonal origin and is diffusely scattered on the peritoneal surface of the abdominopelvic peritoneum, diaphragm, liver, porta hepatis, spleen, and the mesentery of the small bowel and stomach (38). Therefore, debulking surgery is not always optimal for PPC patients. In the series reported by Fromm et al., the rate of successful debulking surgery was only 41% (39). Dubernard et al. obtained a similar rate in their study (9). In this study, only 53.7% of PPSC patients underwent debulking surgery while 31.2% of PPSC patients underwent radical surgery. Interestingly, Kaplan-Meier analysis showed that the OS of radical surgery was similar to or even better than debulking surgery (**Figure 3C**). This study inspired us that radical surgery by removing the spreading organs to minimize the residual tumor may have a better therapeutic effect than debulking surgery. Extensive resection of upper abdominal metastases was recommended for patients who can tolerate this surgery (7, 40, 41). In addition, the research reported by Zhang et al. suggested that if radical resection of tumors becomes very difficult and may result in severe injuries, the operative range should be limited so that postoperative combined chemotherapy could be administered as soon as possible, and finally interval surgery could be an alternative for PPC patients (37).

**Figure 4:**
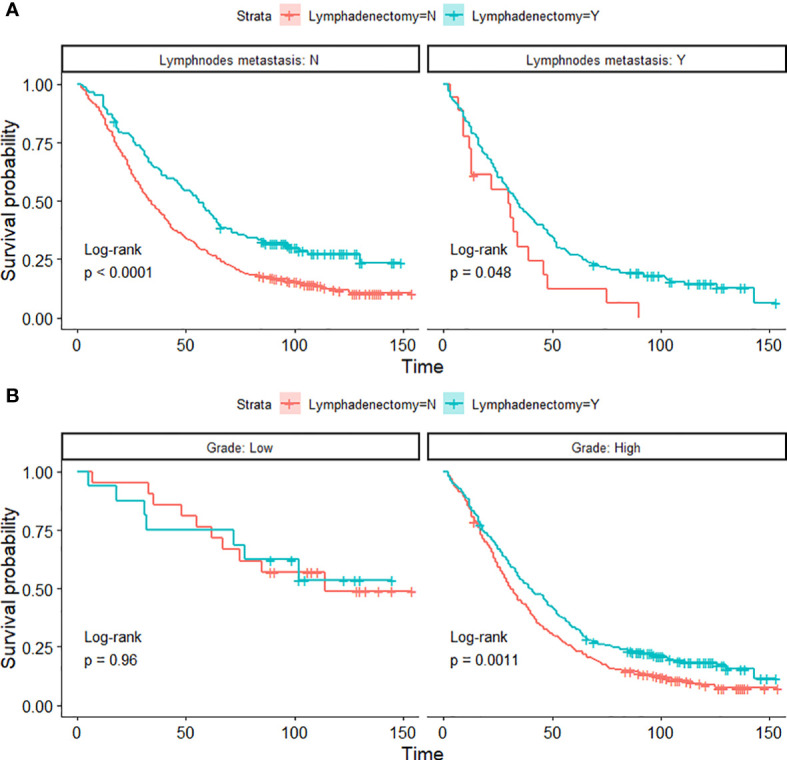
Kaplan-Meier OS curves of lymphadenectomy in lymph node metastasis groups **(A)** and histological-grade groups **(B)** of PPSC patients in the developmental cohort.

The pathological spread of PPSC malignant cells through lymphatic vessels and lymph nodes is not clear (42). Steinhagen et al. reviewed four of the studies and found that they all recommended systematic pelvic and paraaortic lymphadenectomy when an optimal debulking surgery was achieved (42–46). However, there is no consensus on the therapeutic value of systematic lymphadenectomy for PPSC patients and that the prolonged operation may increase the risk for morbidity (43, 47). Although only 27.2% of patients had positive lymphatic metastasis in this study, 45% of patients with lymphadenectomy showed significantly higher OS than those without lymphadenectomy regardless of whether they had lymph node metastasis (**Figures 3D** and **4A**) (*p* < 0.05). What is more, the nomogram suggested that removal of four or more lymph nodes had lower mortality risk than one to three lymph nodes (**Figure 1**). This result may be because the precise nodal status can only be ascertained after complete lymphadenectomy which helps to remove residual lesions more radically. The National Comprehensive Cancer Network (NCCN) guidelines recommended that removal of lymph nodes noted to have potential metastasis at the time of initial diagnosis should be considered, even if not currently suspicious or enlarged (7). In addition, we found that lymphadenectomy did not improve the prognosis of patients with low-grade PPSC while significantly improved the prognosis of patients with high-grade PPSC (**Figure 4B**). Consequently, active lymphadenectomy could significantly improve the prognosis of patients with high-grade PPSC regardless of whether they had lymph node metastasis. More prospective studies are needed to verify this conclusion.

According to the NCCN guidelines (7), the FIGO staging system (8th edition) is used to evaluate the prognosis of ovarian, fallopian tube, and PPC (12). However, different prognosis was observed among patients at the same stage. This prognosis heterogeneity can be explained by its major limitation that disregards other factors, such as age, pathology, marital status, and therapeutic regimen. Using the Kaplan-Meier method, we found that FIGO staging IIIB and IIIC could not distinguish the patient’s prognosis well (**Figure 3E**), and there was less evidence that the cutoff value of 2 cm of macroscopic peritoneal metastasis beyond pelvis had significance of distinguishing the prognosis of PPSC patients. Therefore, we constructed a nomogram that involved these characters to predict individual OS more accurately, proved by the positive NRI, IDI, and LR tests of the nomogram *versus* the FIGO staging system. We made a risk stratification divided into three equal parts named as low, middle, and high risks based on the risk score predicted by the prediction model. The Kaplan-Meier OS curves exhibited significant difference among the three risk groups (**Figure 3F**).

As a retrospective review, there are several limitations to this study, such as incomplete data, a long study period, inconsistent therapies, changing classification standard, and other confounding factors. However, strengths of this study are that this is a large, population-based, long-term review, and analysis of clinicopathological and treatment data of PPSC, and our results suggest that PPSC patients have distinct characteristics with respect to their presentation and survival outcomes. The extensive geographical distribution of patients from population-based cancer registries covering approximately 34.6% of the US population minimizes potential surveillance and selection biases. At the same time, the data of the internal validation and the external validation are both from the SEER database, which may have some impact on its application. Therefore, it is necessary to use other databases for further validation in the future. We followed the recommendation of the transparent reporting of a multivariable prediction model for individual prognosis or diagnosis (TRIPOD) statement to develop and validate the nomogram (48). In addition, we also created a web calculator based on the same clinical prediction model convenient for clinicians to calculate the OS of each patient and formulate individualized therapeutic regimen.

## Conclusion

In view of the higher accuracy, better clinical application effect, and more accurate prognosis prediction compared with the FIGO staging system, our nomogram may be a useful tool to predict prognosis of PPSC patients. Additional research is wanted to further understand the carcinogenesis of PPSC by incorporating translational research with clinical endpoints.

## Data Availability Statement

The datasets presented in this study can be found in online repositories. The names of the repository/repositories and accession number(s) can be found below: https://seer.cancer.gov/data/.

## Author Contributions

The contribution of the individual authors is as follows: MC was involved in data collection, data analysis, and manuscript editing. ZW was involved in data collection, data analysis, and manuscript editing. ZQ was involved in table and figure design. MG was involved in study design and manuscript editing. All authors contributed to the article and approved the submitted version.

## Conflict of Interest

The authors declare that the research was conducted in the absence of any commercial or financial relationships that could be construed as a potential conflict of interest.

## Publisher’s Note

All claims expressed in this article are solely those of the authors and do not necessarily represent those of their affiliated organizations, or those of the publisher, the editors and the reviewers. Any product that may be evaluated in this article, or claim that may be made by its manufacturer, is not guaranteed or endorsed by the publisher.

## Supplementary Material

Supplementary Figure 1 Calibration curves for predicting the 3-, 5-, and 10-year OS of PPSC patients in the training **(A**–**C)**, internal validation **(D**–**F)**, and external validation **(G**, **H)** cohorts.

Supplementary Figure 2 Decision curve analysis of the nomogram and FIGO staging system for predicting the 3-, 5-, and 10-year OS of PPSC patients in the training **(A**–**C)**, internal validation **(D**–**F)**, and external validation **(G**, **H)** cohorts.

Supplementary Figure 3 The 3-, 5-, and 10-year OS of 0.530, 0.330, and 0.137 automatically predicted by the web calculator for a woman of PPSC, aged 60, married, grade high, FIGO IV, lymph node metastasis, debulking surgery, chemotherapy, and lymphadenectomy.
