# Supplementary figures and images for: Development and Validation of Prognostic Nomogram for Primary Peritoneal Serous Carcinoma Compared With FIGO Staging System: A Population-Based Study
Source: Front Oncol. 2021 Aug 19;11:651969. doi: 10.3389/fonc.2021.651969 (PMC8417239; doi:10.3389/fonc.2021.651969)

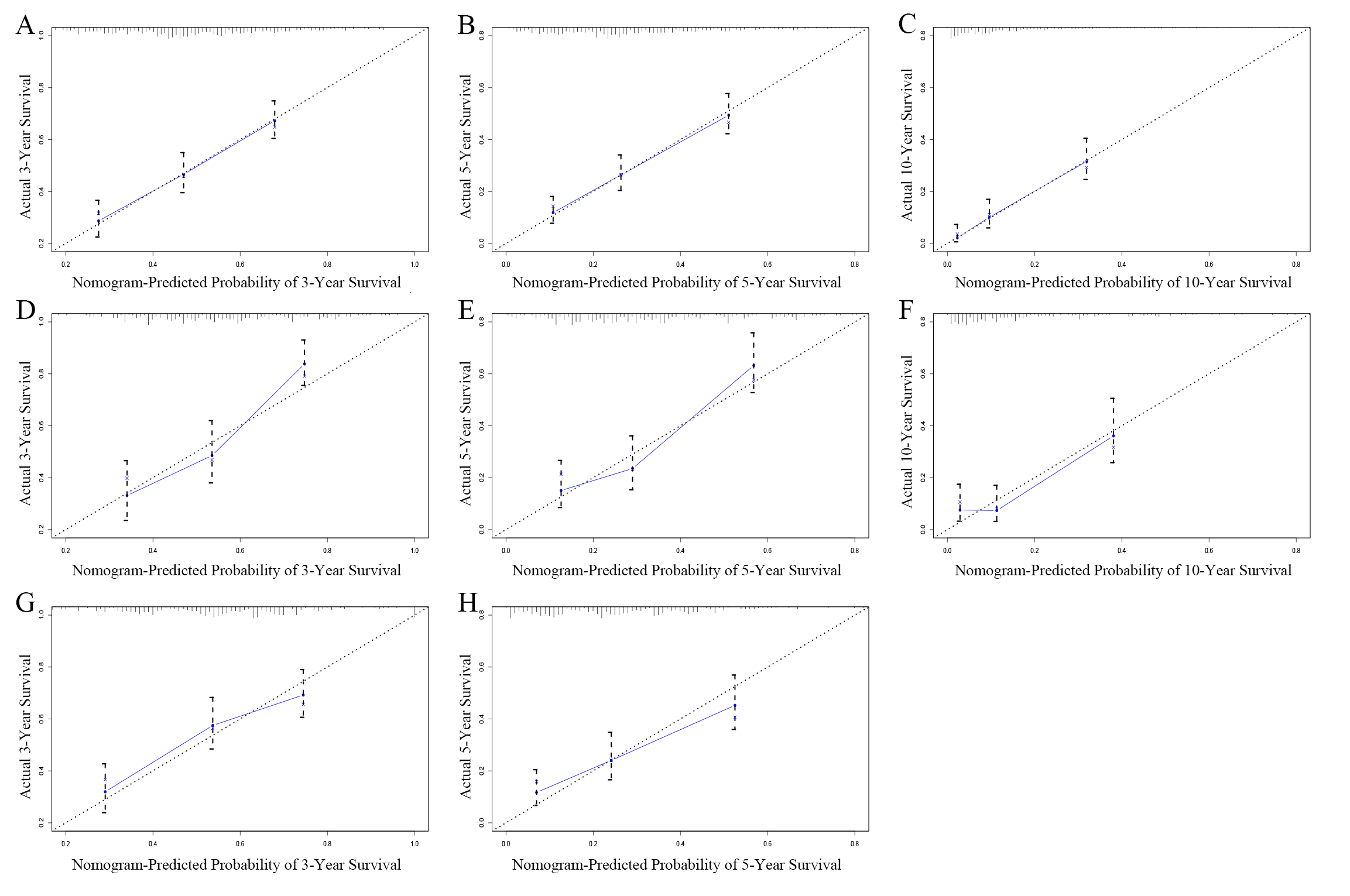

Supplement: Supplementary Figure 1 — Calibration curves for predicting the 3-, 5-, and 10-year OS of PPSC patients in the training (A–C), internal validation (D–F), and external validation (G, H) cohorts. [file Image_1.tif]

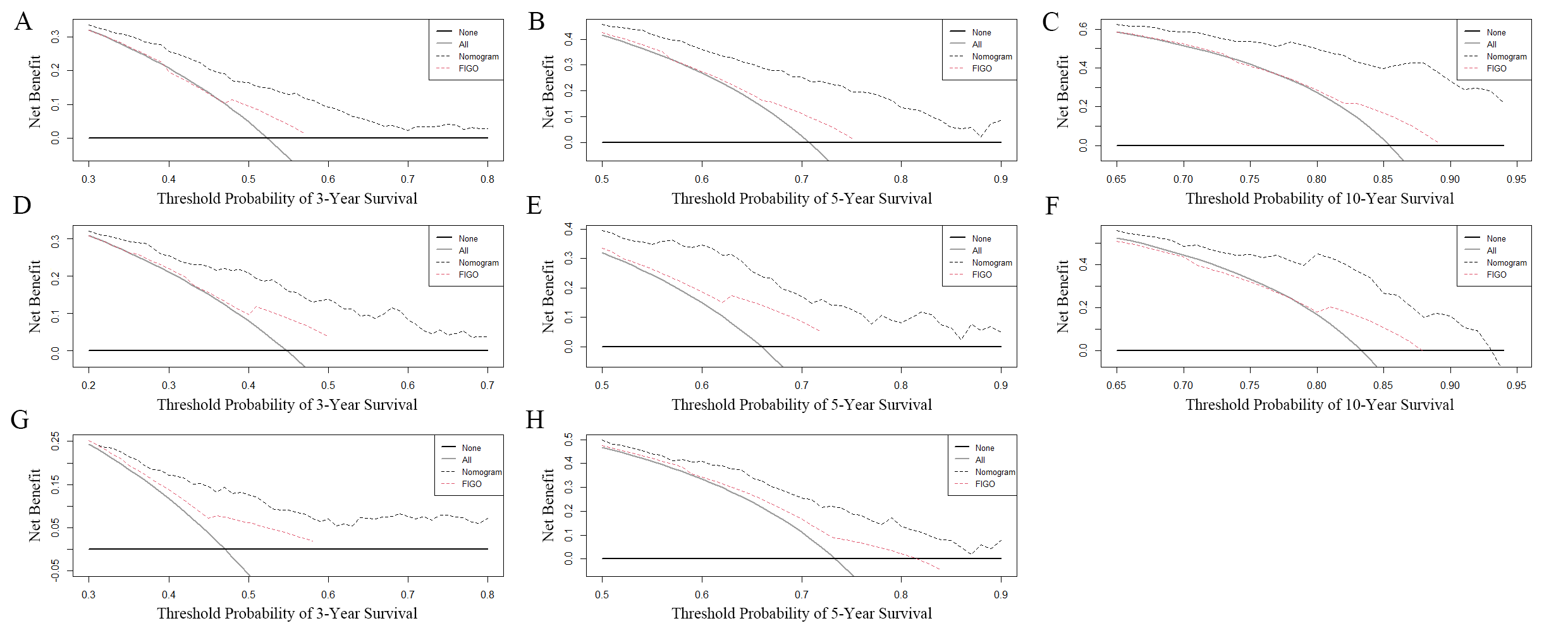

Supplement: Supplementary Figure 2 — Decision curve analysis of the nomogram and FIGO staging system for predicting the 3-, 5-, and 10-year OS of PPSC patients in the training (A–C), internal validation (D–F), and external validation (G, H) cohorts. [file Image_2.tif]

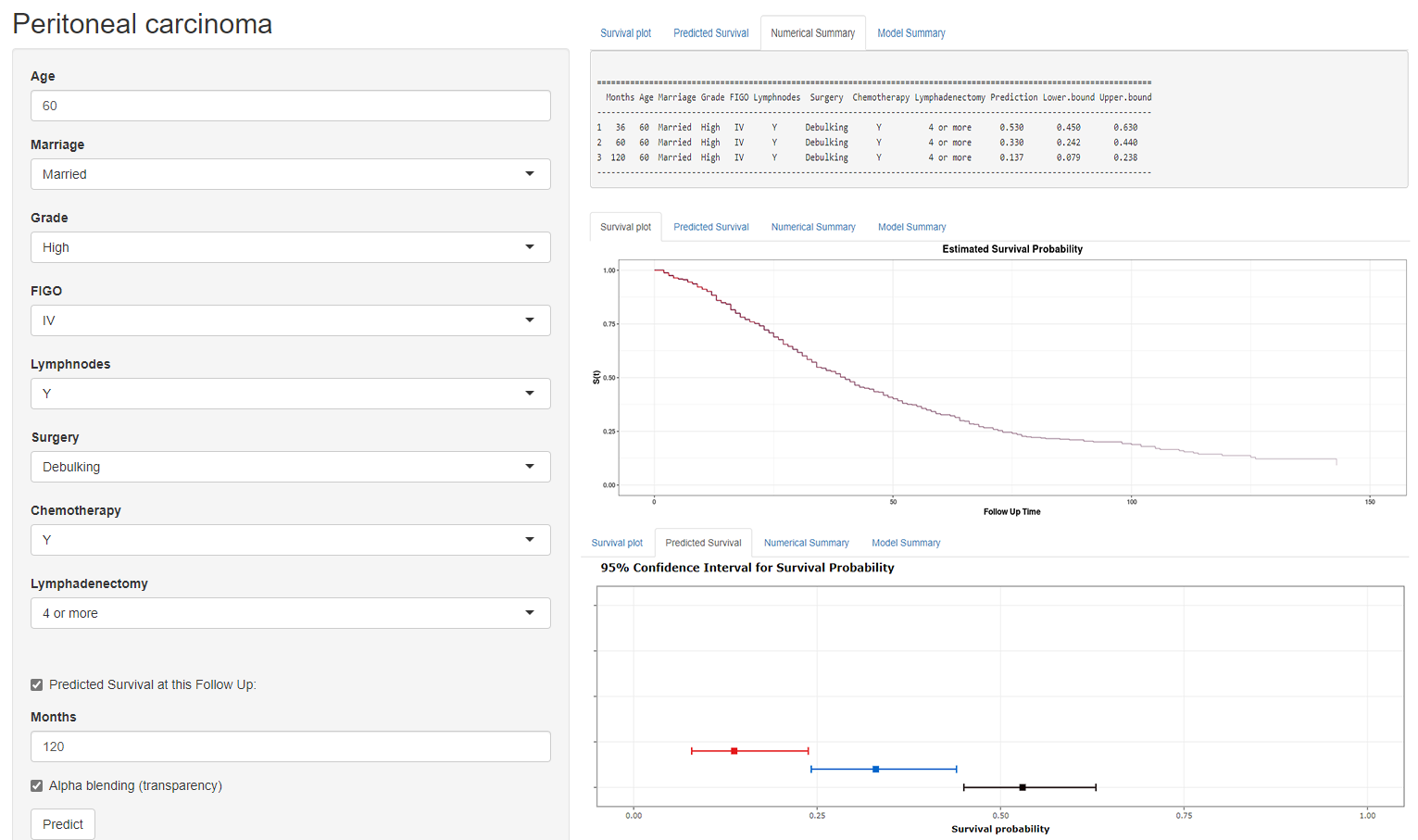

Supplement: Supplementary Figure 3 — The 3-, 5-, and 10-year OS of 0.530, 0.330, and 0.137 automatically predicted by the web calculator for a woman of PPSC, aged 60, married, grade high, FIGO IV, lymph node metastasis, debulking surgery, chemotherapy, and lymphadenectomy. [file Image_3.tif]
