# Supplementary material for: Development and Validation of Prognostic Nomogram for Primary Peritoneal Serous Carcinoma Compared With FIGO Staging System: A Population-Based Study
Source: Front Oncol. 2021 Aug 19;11:651969. doi: 10.3389/fonc.2021.651969 (PMC8417239; doi:10.3389/fonc.2021.651969)
Supplement: Supplementary file 4 [file Table_1.doc]

| **Supplementary Table 1 |** NRI, IDI and Likelihood ratio test of the nomogram and FIGO staging alone in survival prediction for PPSC patients | | | | | | | | | | | | | | | | | | |  |
| --- | --- | --- | --- | --- | --- | --- | --- | --- | --- | --- | --- | --- | --- | --- | --- | --- | --- | --- | --- |
|  | **Training Cohort** | | |  | **Internal Validation Cohort** | | | | | |  | **External Validation Cohort** | | | | | | |  |
| **Index** | **Estimate** | **95% CI** | **P -Val** |  | **Estimate** | | **95% CI** | | **P- Val** | |  | **Estimate** | | | | **95% CI** | | **P- Val** |  |
| **IDI( Nomogram vs FIGO staging)** | | | |  | |  | |  | |  | | |  | |  | |  | |  |
| For 3-year OS | 0.110 | 0.069-0.151 | <0.001 |  | 0.125 | | 0.063-0.192 | | <0.001 | |  | 0.122 | | | | 0.065-0.178 | | <0.001 |  |
| For 5-year OS | 0.139 | 0.085-0.176 | <0.001 |  | 0.139 | | 0.068-0.219 | | <0.001 | |  | 0.103 | | | | 0.029-0.179 | | 0.013 |  |
| For 10-year OS | 0.159 | 0.070-0.231 | <0.001 |  | 0.160 | | 0.064-0.268 | | <0.001 | |  | NA | | | |  | |  |  |
| **NRI( Nomogram vs FIGO staging)** | | | |  | |  | |  | |  | | |  | |  | |  | |  |
| For 3-year OS | 0.240 | 0.146-0.344 | <0.001 |  | 0.310 | | 0.155-0.444 | | <0.001 | |  | 0.227 | | | | 0.109-0.349 | | 0.007 |  |
| For 5-year OS | 0.294 | 0.164-0.382 | <0.001 |  | 0.309 | | 0.134-0.438 | | <0.001 | |  | 0.171 | | | | 0.002-0.349 | | 0.027 |  |
| For 10-year OS | 0.502 | 0.312-0.648 | <0.001 |  | 0.386 | | 0.145-0.599 | | <0.001 | |  | NA | | | |  | |  |  |
| **Likelihood ratio test** | |  |  |  | |  |  | |  | |  | | |  | |  | |  |  |
| Nomogram | -2184.9 |  |  |  | -757.3 | |  | |  | |  | -1038.0 | | | |  | |  |  |
| FIGO | -2236.4 |  |  |  | -780.9 | |  | |  | |  | -1076.9 | | | |  | |  |  |
| χ^2^ | 103.0 |  | <0.001 |  | 47.2 | |  | | <0.001 | |  | 77.8 | | | |  | | <0.001 |  |
| FIGO, the International Federation of Gynecology and Obstetrics; NA, Not Available. | | | | | | | | | | | | | | | | | | |  |
